# Mechanochemical Synthesis of CuO/MgAl_2_O_4_ and MgFe_2_O_4_ Spinels for Vanillin Production from Isoeugenol and Vanillyl Alcohol

**DOI:** 10.3390/molecules24142597

**Published:** 2019-07-17

**Authors:** Behgam Rahmanivahid, Maria Pinilla-de Dios, Mohammad Haghighi, Rafael Luque

**Affiliations:** 1 Esfarayen University of Technology, Esfarayen 96619-98195, Iran; 2 Departamento de Química Orgánica, Facultad de Ciencias, Universidad de Córdoba, Campus de Rabanales, Edificio Marie Curie (C-3), Ctra Nnal IV-A, Km 396, E14014 Córdoba, Spain; 3 Chemical Engineering Faculty, Sahand University of Technology, P.O. Box 51335-1996, Sahand New Town, Tabriz 53318-11111, Iran; 4 Peoples Friendship University of Russia (RUDN University), 6 Miklukho Maklaya str., 117198 Moscow, Russia

**Keywords:** high speed ball-milling, mechanochemistry, spinel, vanillin, vanillyl alcohol, isoeugenol

## Abstract

CuO/MgAl_2_O_4_ and CuO/MgFe_2_O_4_ catalysts were successfully synthesized with the use of spinel supports by a very simple and low-cost mechanochemical method. High-speed ball-milling was used to synthesize these catalyst supports for the first time. Materials were subsequently characterized by using XRD, FESEM, TEM, EDS-Dot mapping, XPS, BET-BJH, and Magnetic Susceptibility to investigate the physical-chemical characteristics of the catalysts. Acidity evaluation results indicated that the catalyst with the Mg-Al spinel support had more acid sites. XRD results showed a successful synthesis of the catalysts with large crystal sizes. Both catalysts were used in isoeugenol oxidation and vanillyl alcohol to vanillin reactions, with the CuO/MgAl_2_O_4_ showing optimum results. This catalyst provided 67% conversion (74% selectivity) after 2 h and this value improved to 81% (selectivity 100%) with the second reaction after 8 h. The CuO/MgFe_2_O_4_ catalyst in the first reaction after five hours revealed 53% conversion (47% selectivity) and after eight hours with the second reaction, the conversion value improved to 64% (100% selectivity). In terms of reusability, CuO/MgAl_2_O_4_ showed better results than the CuO/MgFe_2_O_4_ catalyst, for both reactions.

## 1. Introduction

Vanillin is a chemical compound from the aromatic aldehydes family with many industrial applications [1]. This valuable substance is used in perfumes, food, cosmetics, pharmaceuticals, and other chemical industries [2,3,4]. Vanillin naturally exists in some plant families which have been used for extracting it in so many different ways [5]. According to the multiple uses of vanillin, the annual required amount of this material has been estimated at 12,000 tons. Nowadays, due to the lack of natural resources (less than 1% is provided from them) and considering the low price of synthetic vanillin, this substance is produced by industrial methods [6,7]. There are various industrial methods to produce vanillin. One of them is the bioconversion of materials such as vanillyl alcohol, vanillic acid, creosol, phenolic stilbenes, glucose, lignin, isoeugenol, and eugenol [8,9,10]. Another method to produce vanillin is using biocatalysts because the marginal cost of the product then becomes very low [11,12,13,14]. Chemical synthesis of vanillin from lignin, guaiacol, coniferin, ferulic acid as raw materials is also an important way to produce this substance [3,15,16]. In recent years, vanillin production by the oxidation of some materials such as isoeugenol and vanillyl alcohol has been considered [7,17,18]. In this method, raw materials with an oxidizer such as hydrogen peroxide in the presence of a catalyst with the ability of oxidation produce vanillin [19]. There are several catalysts with oxidation ability which are used in various industries. Some of these catalysts such as Cu-Ti composite, Mn-doped ceria, and Co_3_O_4_ were used in the vanillin production reaction [17,18,19]. However, these catalysts need high temperature and pressure or a significant time for this reaction. Also other catalysts such as Pd [20,21], Mn_2_O_3_ [22,23,24], and Au [25,26,27] have been used in oxidation reactions of CH_4_, CO, NO, and CH_4_O. In several references, CuO was used as a powerful catalyst for oxidation reactions [28,29,30,31]. It seems that these metal oxides, due to their low cost and high oxidation power, are suitable catalysts for the oxidation of isoeugenol and vanillyl alcohol. In this work, CuO was employed as active phase for the synthesis of CuO-based catalysts in view of their application in the selective oxidation of isoeugenol to vanillin. Mg-Al and Mg-Fe spinels (MgAl_2_O_4_ and MgFe_2_O_4_) were selected as supports due to their previously reported convenient catalytic properties [32,33,34,35].

In recent years, mechanochemistry emerged as a relevant methodology for the synthesis, preparation, and design of nanomaterials [36]. MgFe_2_O_4_ and MgAl_2_O_4_ spinels were previously synthesized via a mechanochemical method by other researchers, but, with low speed, lengthy, and energy intensive ball-milling conditions (e.g., over 12 h) [37,38]. In this work, high speed ball-milling mechanochemistry was employed as a simple and low-cost methodology, for the first time, in the synthesis of these spinels.

Various analyses, such as X-ray Powder Diffraction (XRD), Field Emission Scanning Electron Microscopy (FESEM), Transmission Electron Microscopy (TEM), BET Surface Area and BJH Pore Size, Energy Dispersive X-Ray Spectroscopy (EDS)-Dot mapping, X-Ray Photoelectron Spectroscopy (XPS), and Magnetic Susceptibility, were used to determine the characteristics of the synthesized catalysts.

## 2. Results and Discussion

### 2.1. Characterization of the Nanocatalysts

#### 2.1.1. XRD Analysis

**Figure 1:**
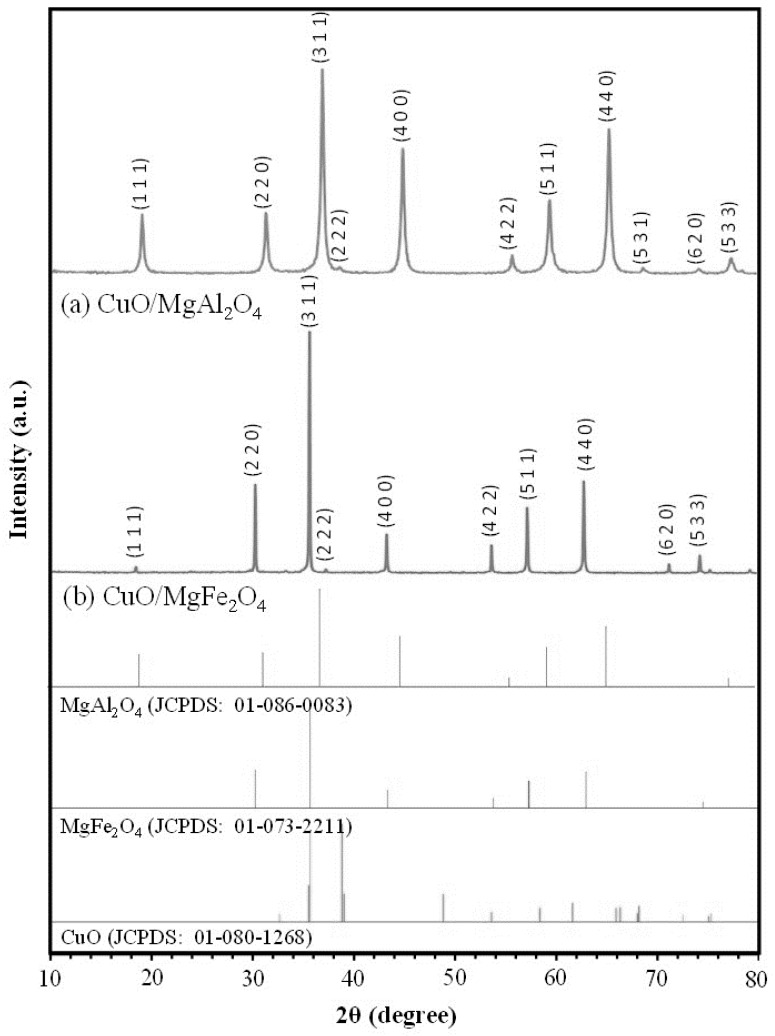
XRD patterns of prepared nanocatalysts: (**a**) CuO/MgAl_2_O_4_, (**b**) CuO/MgFe_2_O_4__._

XRD analyses of the synthesized samples are shown in Figure 1. Comparing the analysis of the CuO/MgAl_2_O_4_ sample with the standard peaks of MgAl_2_O_4_ spinels (JCPDS: 01-086-0083; Cubic, 2θ = 19.0, 31.3, 36.8, 44.8, 55.6, 59.3, 65.2, and 77.3), the successful synthesis of this spinel as catalyst support could be easily recognized. However, comparing the analysis of this sample with the standard patterns of CuO (JCPDS: 01-080-1268; 2θ = 35.5, 35.7, 38.8, 39.0, 48.7, 53.5, 58.4, 61.6, 65.9, 66.3, and 68.2), no significant peak was observed in these samples. 

This phenomenon represents an appropriate and uniform distribution also with small particles of CuO on the surface of the catalyst support [32]. This occurs due to the use of the mechanochemical method in the distribution of the active phase on the catalyst support. Furthermore, peaks of CuO/MgFe_2_O_4_ corresponding to the standard patterns of MgFe_2_O_4_ (JCPDS: 01-073-2211; Cubic, 2θ = 30.2, 35.6, 43.2, 53.6, 57.2, 62.8, and 74.3) were observed, which confirms the successful synthesis of this spinel. Also, studying the analysis of this sample and standard peaks of CuO, no peak was observed. This phenomenon can be attributed to the low ratio of the active phase to the support (0.5 wt%), to the proper and uniform dispersion, and also to the small particle size due to the use of a mechanochemical method. Crystal sizes of these two samples are shown in Table 1, which are calculated by the Scherrer equation [39]. These results showed sizes of 25.9 nm and 78 nm for the CuO/MgAl_2_O_4_ and CuO/MgFe_2_O_4_ samples, respectively. Larger crystal sizes of the synthesized sample with magnetic support indicate that this sample has a lower surface size than the other sample. 

#### 2.1.2. TEM and SEM Analysis

**Figure 2:**
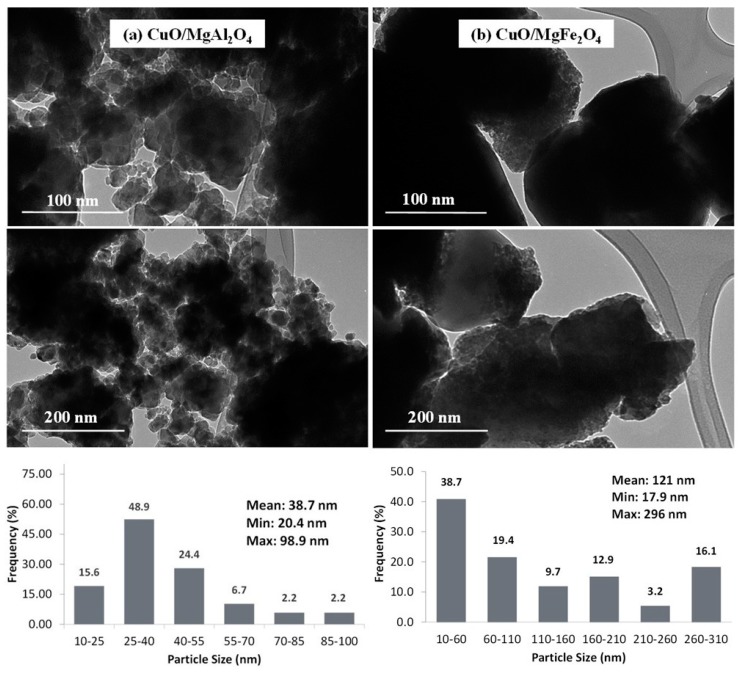
TEM images and size distribution of (**a**) CuO/MgAl_2_O_4_ catalyst, (**b**) CuO/MgFe_2_O_4_ catalyst.

TEM analyses of the synthesized samples are shown in Figure 2. TEM images of CuO/MgAl_2_O_4_ (Figure 2a) indicate that the average particle size of this catalyst is smaller than 100 nm and, with more detail, crystalline forms of the particles can be seen. On the contrary, the analysis of CuO/MgFe_2_O_4_ (Figure 2b) shows that the particle size is around 200 nm and also the formation of large crystals can be seen in this sample. The minimum, maximum, and average particle size of both samples are shown in a particle size distribution plot. The average particle sizes obtained of CuO/MgAl_2_O_4_ and CuO/MgFe_2_O_4_ were 39 nm and 121 nm, respectively, which is in good agreement with results of crystal size.

**Figure 3:**
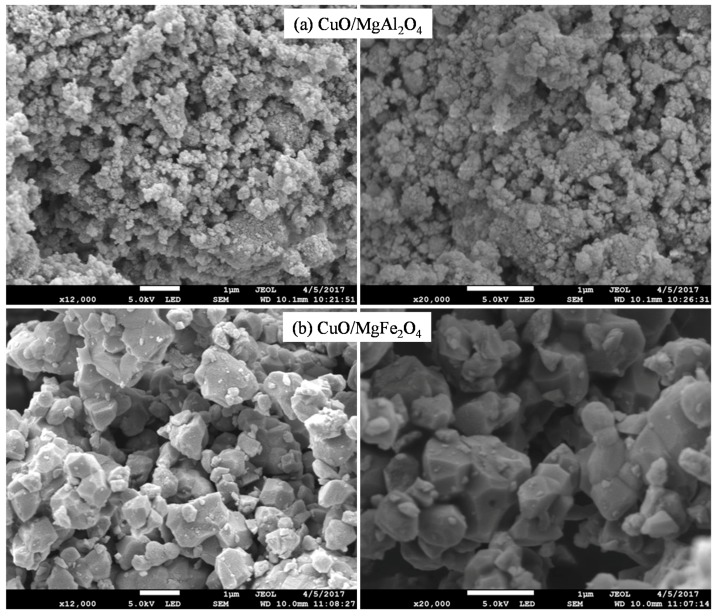
SEM images of synthesized nanocatalysts: (**a**) CuO/MgAl_2_O_4_, (**b**) CuO/MgFe_2_O_4__._

Due to the magnetic properties of MgFe_2_O_4_, the associated TEM images are not more transparent than the other sample. SEM analysis of CuO/MgAl_2_O_4_ and CuO/MgFe_2_O_4_ catalysts which are shown in Figure 3 confirm the results of TEM analysis. These analyses show that the particles of CuO/MgAl_2_O_4_ (Figure 3a) are smaller than the particles of CuO/MgFe_2_O_4_ (Figure 3b). Particle sizes with a magnetic support are large so the crystal regular form can be easily observed. 

Both catalysts were found to be non-porous materials. However, due to the small particle size of the CuO/MgAl_2_O_4_ catalyst, it can be predicted that its surface area is higher than the other catalyst area. It is noteworthy that the results of this analysis confirmed the XRD analysis results.

#### 2.1.3. EDX Analysis

**Figure 4:**
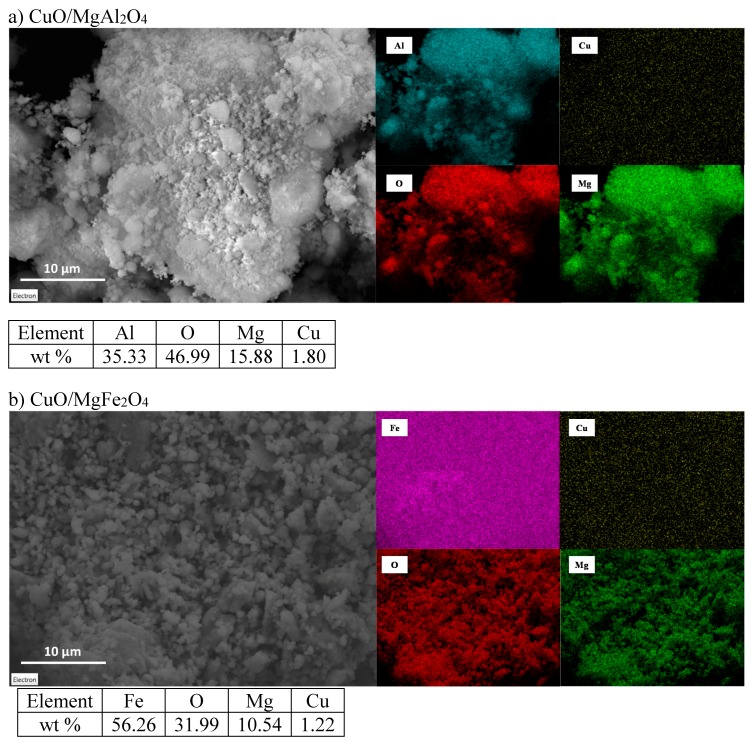
EDX elemental dot mapping analysis of (**a**) CuO/MgAl_2_O_4_ and (**b**) CuO/MgFe_2_O_4_ nanocatalysts.

EDX analysis was carried out to determine the distribution of the elements on each catalyst surface and its impact on the activity of the catalysts in the reaction [40,41]. EDX-dot mapping analysis was used for both CuO/MgAl_2_O_4_ and CuO/MgFe_2_O_4_ catalysts, respectively. Results are shown in Figure 4. Mg, Al, and O elements follow a uniform distribution in the CuO/MgAl_2_O_4_ sample which results in a proper and uniform formation of this spinel. Dot mapping of Cu element in this catalyst shows a highly proper and uniform distribution of CuO on the support surface, as the active phase. On the other hand, CuO/MgFe_2_O_4_ catalyst follows a uniform distribution of Mg, Fe, and O elements and shows also a good distribution of Cu on the support surface. Both element weight distributions are consistent with the theoretical data. It can be said that these uniform distributions are attributable to the mechanochemical synthesis method. This method forces a proper mix of the components of the spinel support synthesis and also an appropriate distribution of the active phase (CuO) on the supports (MgAl_2_O_4_ and MgFe_2_O_4_) [42,43].

#### 2.1.4. XPS Analysis

**Figure 5:**
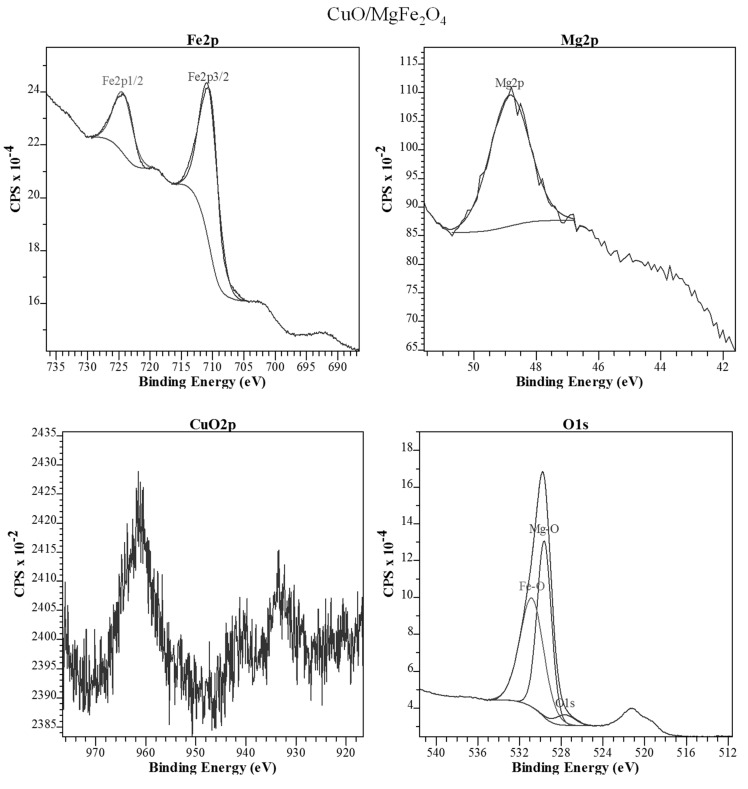
XPS data of CuO/MgFe_2_O_4_ nanocatalyst.

**Figure 6:**
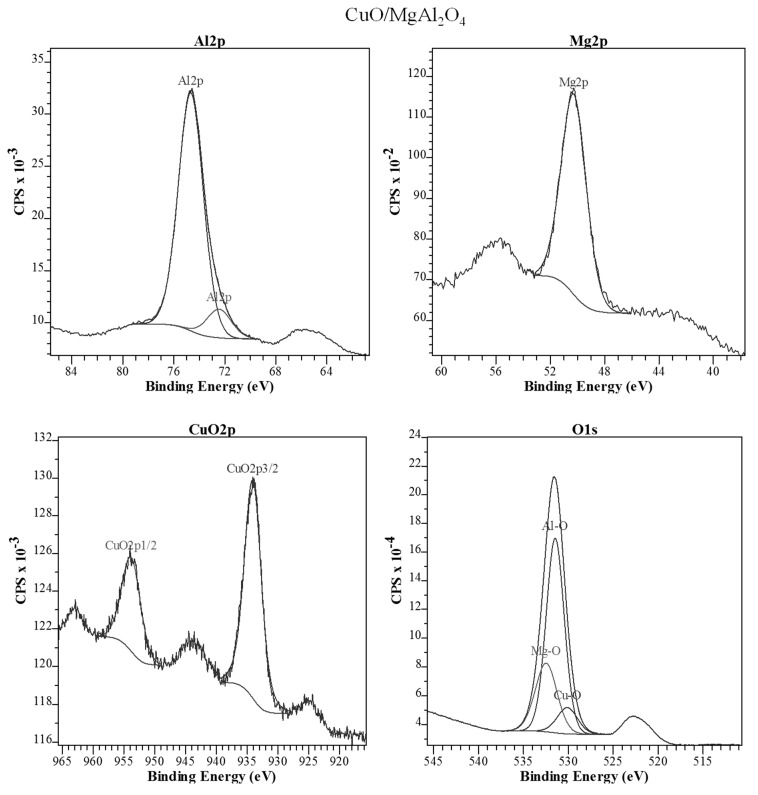
XPS data of CuO/MgAl_2_O_4_ nanocatalyst.

The surfaces of both synthesized samples were studied by XPS analysis and the results are shown in Figure 5 and Figure 6. Before analysis, the peak locations for both samples were calibrated according to the peak of C1s (adsorbed species CO and CO_2_) which appears at 284.6 eV. Figure 5 shows peaks at 724.6 and 711.1 eV which are related to Fe2p_3/2_ and Fe2p_1/2_ respectively. They confirmed the existence of Fe^3+^ species in the CuO/MgFe_2_O_4_ catalyst. 

The presence of the Mg2p peak at 48.9 eV is related to Mg^2+^ in the sample. So, it is possible to conclude the successful formation of MgFe_2_O_4_ spinel [44,45,46]. The O1s peak at 529.8 eV belongs to the Mg–O, Fe–O bond or is related to absorbed oxygen of various species on the catalyst surface [44,45,46]. In this case, the peak of CuO was not observed correctly which may be due to the very low amount (0.5 wt%) and also XPS is a local/superficial analysis [47].

The XPS image corresponding to the CuO/MgAl_2_O_4_ catalyst is shown in Figure 6. Peaks at 50.3 eV and 74.7 eV which are related to Mg2p and Al2p, indicate the presence of Mg^2+^ and Al^3+^ species in this sample which proves the successful synthesis of MgAl_2_O_4_ spinel [47,48,49]. Also various species of O1s detected in the synthesized samples at 531.5 and 532.5 eV peaks are related to Al–O and Mg–O bonds, respectively and also the peak at 530.2 eV, because of the oxygen of absorbed species on the catalyst surface or it is related to Cu–O bond in the active phase [49,50]. For Cu^2+^ species, the peaks at 934.0 and 954.0 eV are related to Cu2p in this sample which shows the existence of CuO on the catalyst surface [51]. It should be noted that a partial change in peak locations of some species, with decreasing or increasing bonding energy, is due to a change in the energy level of various species in different environments [45,52,53,54]. Results from this analysis support the results from XRD and EDS analysis.

#### 2.1.5. BET-BJH Analysis

**Figure 7:**
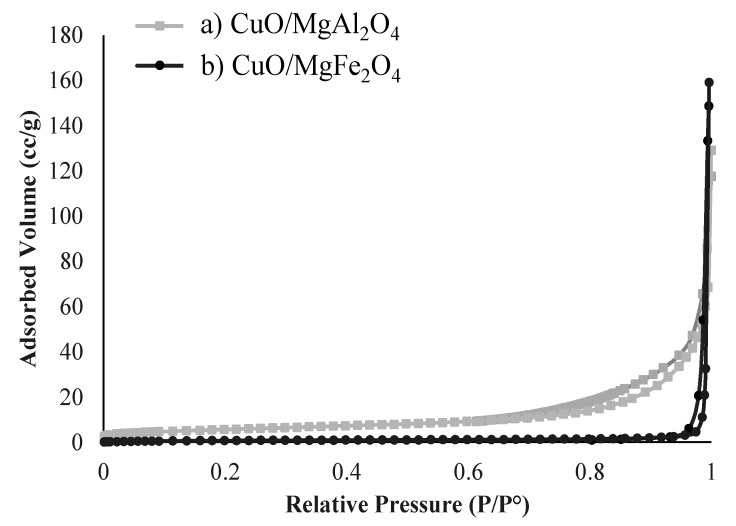
Adsorption/desorption isotherms of the nanocatalysts (**a**) CuO/MgAl_2_O_4_ and (**b**) CuO/MgFe_2_O_4__._

The surface area and pore diameter of the catalyst are two key characteristics to be evaluated in all catalysts. Synthesized catalysts were evaluated by BET-BJH analysis and the results are shown in Table 1 and Figure 7. According to the table, the surface areas of CuO/MgAl_2_O_4_ and CuO/MgFe_2_O_4_ catalysts were 20 m^2^/g and 3 m^2^/g, respectively. The reason for the very small surface area of the CuO/MgFe_2_O_4_ catalyst is its large particle and crystal size which is demonstrated in XRD and FESEM analysis. 

From Figure 7, which shows the adsorption and desorption isotherms of the synthesized samples, these catalysts can be easily categorized into type III of the IUPAC classification. The hysteresis forms of the samples indicate that both synthesized catalysts are non-porous and have plate structures, so the samples have an inter-particle surface. However, according to Table 1, the obtained pore diameters for CuO/MgAl_2_O_4_ and CuO/MgFe_2_O_4_ catalysts are 51.4 nm and 18.2 nm, respectively. These results confirm previous analyses, such as XRD and SEM.

#### 2.1.6. Acidity

Pyridine (PY) and 2,6-dimethylpyridine (DMPY) absorption methods were used at 200 °C (pulse chromatographic titration methodology) to investigate the acidity of CuO/MgAl_2_O_4_ and CuO/MgFe_2_O_4_ catalysts [55,56,57]. Through the PY absorption, it is possible to obtain the total amount of acid sites. By the DMPY absorption method, Brönsted acid sites can be determined. Lewis acid sites are calculated by subtracting these values from each other [58]. The results of this method can be seen in Table 2. According to the results, the total amount of acid sites of the CuO/MgAl_2_O_4_ catalyst (69 μmol PY/g) is higher than the amount of the CuO/MgFe_2_O_4_ catalyst (38 μmol PY/g). However, the amount of Lewis acid sites is almost the same for both samples (26 μmol PY/g). The reason for the difference in the value of Brönsted acid sites and the coincidence on the value of Lewis acid sites is the magnetic support of MgFe_2_O_4_ which is related with the surface area. The effect of acid sites was further determined by examining the catalyst activity.

#### 2.1.7. Magnetic Susceptibility Analysis

Magnetic properties of CuO/MgAl_2_O_4_ and CuO/MgFe_2_O_4_ catalysts are shown in Table 2. Due to the use of Mg-Fe spinel in the synthesis of CuO/MgFe_2_O_4_ as catalyst support, this sample showed a highly desirable magnetic susceptibility of 416 × 10^−6^ m^3^/kg. On the contrary, the synthesized catalyst with Mg-Al spinel does not have magnetic susceptibility. According to the magnetic behavior of the CuO/MgFe_2_O_4_ catalyst, this sample has a great ability to be separated easily from the reaction mixture which can be considered an advantage for this catalyst.

### 2.2. Catalytic Performance Study toward Vanillin Production

**Figure 8:**
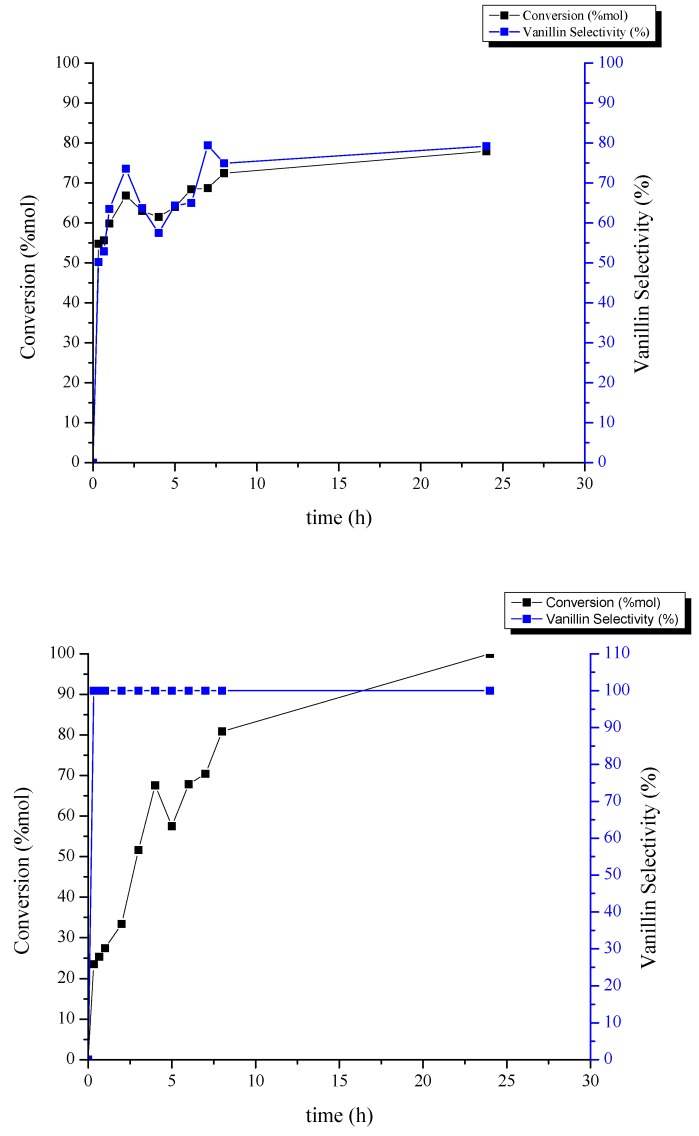
Catalytic activity at 90 °C of CuO/MgAl_2_O_4_ catalyst in the oxidation of isoeugenol to vanillin (**top image**) and CuO/MgAl_2_O_4_ catalyst in the oxidation of vanillyl alcohol to vanillin (**bottom image**).

**Figure 9:**
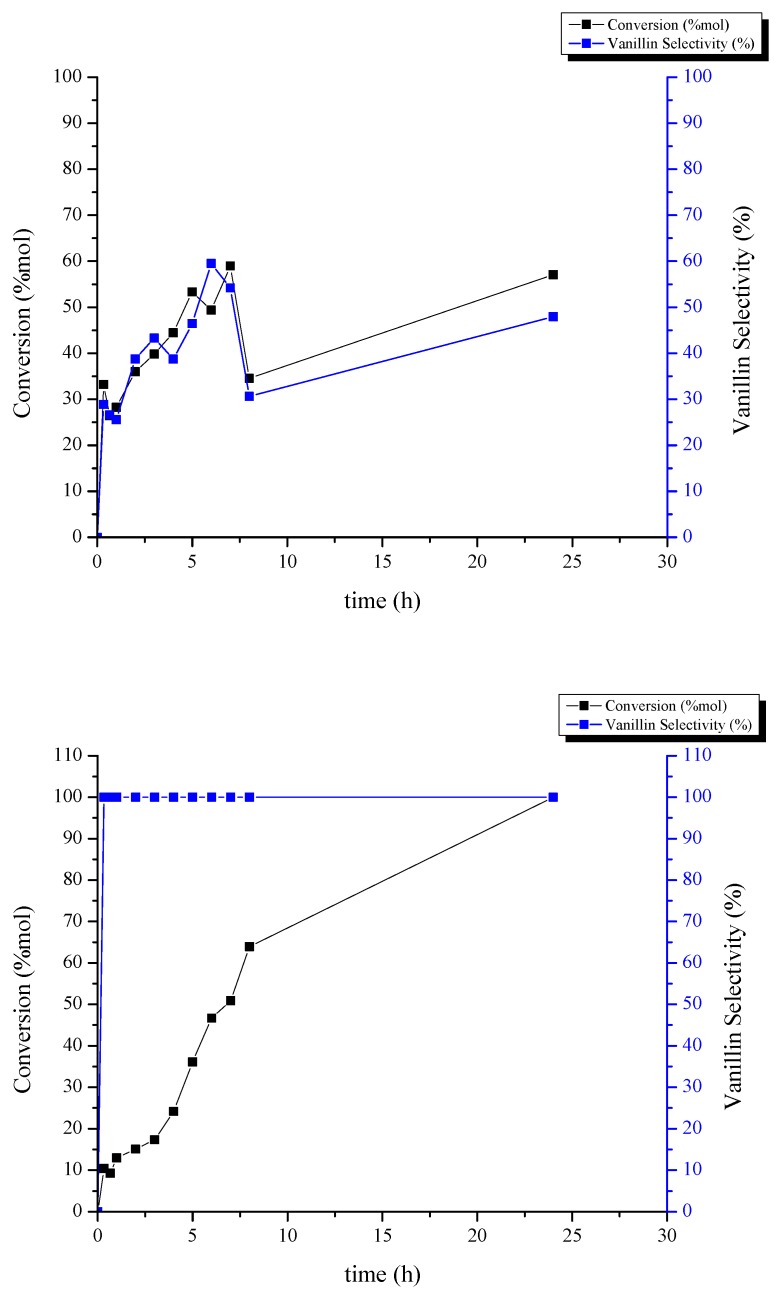
Catalytic activity at 40 °C of CuO/MgFe_2_O_4_ catalyst in the oxidation of isoeugenol to vanillin (**top image**) and CuO/MgFe_2_O_4_ catalyst in the oxidation of vanillyl alcohol to vanillin (**bottom image**).

In order to evaluate the synthesized catalysts activity, CuO-based spinel systems were employed in oxidation reactions of isoeugenol and vanillyl alcohol to vanillin. To carry out these reactions, an oxidizer (hydrogen peroxide) and a solvent (acetonitrile) were used. Both reactions were carried out at a temperature of 90 °C. After using GC to obtain the amount of the reaction products, the conversion and selectivity of each reaction were calculated. Results are shown in Figure 8 and Figure 9. To determine the behavior of each catalyst in the reaction, first both vanillin production reactions were performed at 90 °C (isoeugenol oxidation) and 40 °C (vanillyl alcohol oxidation) over 24 h without any catalyst (Table 3 and Table 4, respectively). Using the synthesized catalysts, the results showed that CuO/MgAl_2_O_4_ catalyst has a very good activity in the isoeugenol oxidation reaction. After two hours of reaction, conversion reached 67%. The selectivity of vanillin production was 74%, at the same time. After 8 h with the same catalyst, conversion was 81% and the selectivity in the oxidation reaction of vanillyl alcohol to vanillin was raised to 100%. The performance of CuO/MgFe_2_O_4_ was not so efficient. With this catalyst, after 2 h, the conversion was 36% with a selectivity of 39% in the vanillin production reaction from isoeugenol. After 5 h, the conversion increased to 53% with a selectivity of 46%. After 8 h of the oxidation reaction of vanillyl alcohol, the conversion was 64% with a selectivity of 100%. 

Taking into account the results, it is possible to determine that the Mg-Al spinel support exhibited a comparably superior performance to that of Mg-Fe spinel. This may be due to the greater surface area and pore volume of CuO/MgAl_2_O_4_. Active sites for this reaction were previously reported to be moderate acidic and/or redox metal sites, in this case being a synergistic combination between the CuO phase and the spinel support. Another important characteristic of heterogeneous catalysts is their reusability. The CuO/MgAl_2_O_4_ catalyst was used four consecutive times in the isoeugenol and vanillyl alcohol oxidation reaction over 2 and 8 h, respectively and CuO/MgFe_2_O_4_ catalyst was used over 5 and 8 h. For this purpose, CuO/MgAl_2_O_4_ and CuO/MgFe_2_O_4_ catalysts were separated once finished from the reaction mixture with filter paper and by a magnet, respectively. Then, they were oven-dried for 24 h at 110 °C to use again in reactions. From Figure 9, the first reuse of CuO/MgAl_2_O_4_ catalyst in the vanillin production reaction from isoeugenol, showed a good performance with a 7% decrease in the conversion and a 13% reduction in selectivity. After the second and third reuse, the conversion changed to 40% and 29%, respectively and the selectivity varied to 42% for the second reuse and 28% for the third one. With the same catalyst, in the oxidation reaction of vanillyl alcohol the conversion gradually decreased to 79% after the first reuse. 

For the next iterations, conversion decreased to 56% and 29%, respectively with 100% selectivity. On the other hand, the CuO/MgFe_2_O_4_ catalyst showed different results. In both reactions, drastic reductions were observed after their reuse. The conversion of this catalyst in the isoeugenol oxidation reaction after the first reuse was 29% with a selectivity of 23%. After the second and third iterations the conversion was almost constant at 17% with a selectivity of 8%. The conversion of this catalyst after the first reuse in the vanillyl alcohol oxidation reaction was 42%. This value decreased to 26% and 25% after the second and third reuse, respectively. According to the results, it is possible to conclude that both catalysts CuO/MgAl_2_O_4_ and CuO/MgFe_2_O_4_ have good ability to produce vanillin from vanillyl alcohol and isoeugenol. Based on the analysis and the values from conversion, selectivity and reusability, it can be said that the MgAl_2_O_4_ spinel is the most appropriate support for the active phase of CuO to perform both reactions.

## 3. Conclusions

CuO/MgAl_2_O_4_ and CuO/MgFe_2_O_4_ catalysts with spinel supports were synthesized for the first time by using a mechanochemical method (high-speed ball-milling) as a quick and efficient method. The active phase of CuO was distributed on the catalyst surface using conventional (low speed) ball milling. With this method, the synthesis time and the costs were significantly reduced. Results from different analyses showed that CuO/MgFe_2_O_4_ catalyst had higher particle and crystal size than CuO/MgAl_2_O_4_ catalyst which is related to the small surface area. Larger particle size caused a smaller area. Both catalysts used in the oxidation reaction of isoeugenol and vanillyl alcohol showed a good ability to produce vanillin. CuO/MgFe_2_O_4_ catalyst showed high magnetic susceptibility and therefore easy separation from the reaction mixture. In both reactions comparing the conversion, selectivity, and reusability of the catalysts, CuO/MgAl_2_O_4_ presented better characteristics for these reactions, explainable because of its surface area and acid sites.

## 4. Materials and Methods

### 4.1. Materials

Magnesium oxide (MgO 99%, Aldrich, St. Louis, MO, USA), aluminum oxide (Al_2_O_3_ 99%, Panreac, Barcelona, Spain), iron oxide III (α-Fe_2_O_3_ 99%, Merck, Kenilworth, NJ, USA) and hydrated copper chloride (CuCl_2_.2H_2_O 99.8%, Sigma-Aldrich, St. Louis, MO, USA) were used to synthesize CuO/MgAl_2_O_4_ and CuO/MgFe_2_O_4_. Isoeugenol (C_10_H_12_O_2_ 98%, Aldrich), acetonitrile (C_2_H_3_N 99.8%, Panreac), vanillyl alcohol (C_8_H_10_O_3_ 98%, Aldrich), and hydrogen peroxide (H_2_O_2_ 50 wt% in H_2_O, Sigma-Aldrich) were used for vanillin production reactions. All reagents were used without further purification.

### 4.2. Preparation and Procedure of the Nanocatalysts

**Figure 10:**
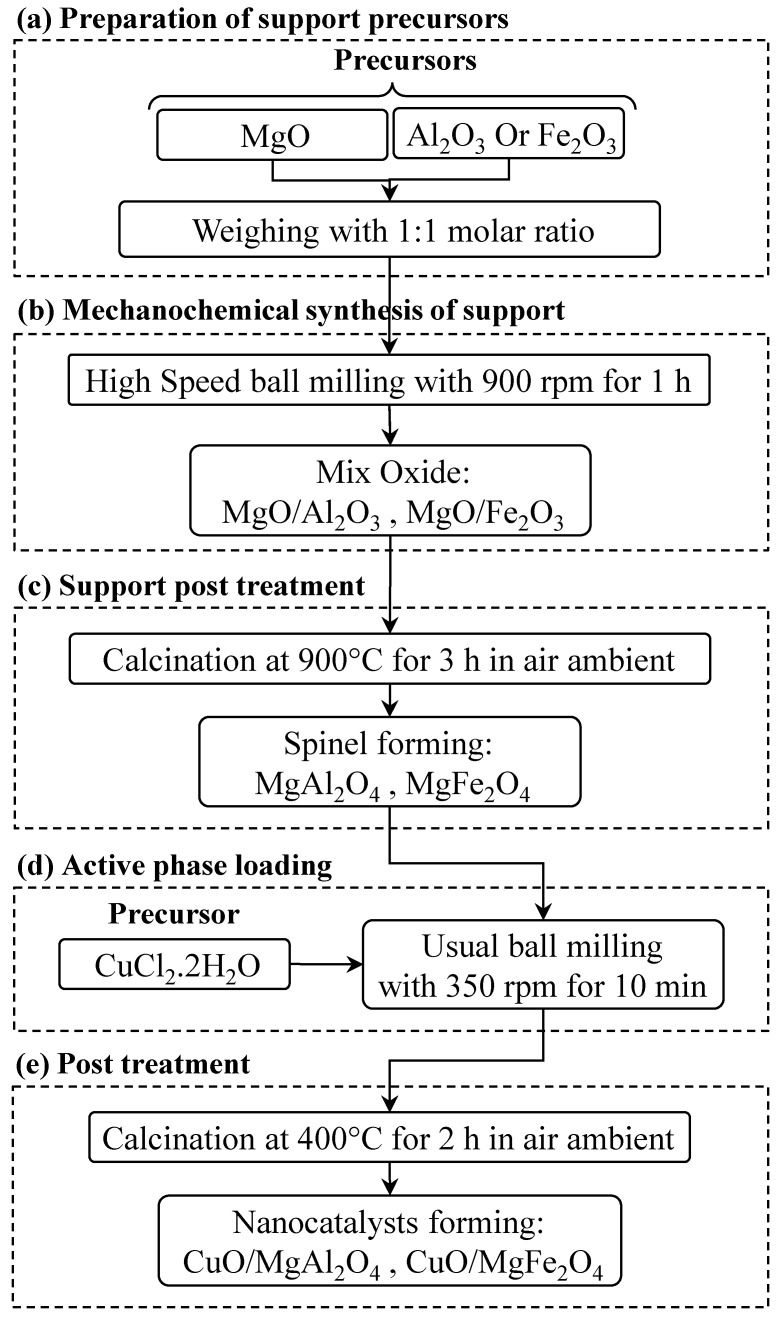
Mechanochemical synthesis of CuO/MgAl_2_O_4_ and CuO/MgFe_2_O_4_ nanocatalysts.

The synthesis procedure of CuO/MgAl_2_O_4_ and CuO/MgFe_2_O_4_ nanocatalysts is shown in Figure 10. As can be seen, for the synthesis of MgAl_2_O_4_ spinel, stoichiometric amounts of MgO and Al_2_O_3_ were mixed by high speed ball milling (E_max_ model, Retsch, Haan, Germany) at 900 rpm for 1 h. Then, the obtained mixture of oxides was calcined in a furnace with air flow at 900 °C for 3 h. After preparing the spinel support for the deposition of CuO active phase on its surface, specific amounts of hydrated copper chloride and MgAl_2_O_4_ (to achieve a 0.5 wt% CuO in the final material) were mixed by conventional ball milling (PM 100 model, Retsch, Germany) at 350 rpm for 10 min. Finally, to synthesize the CuO/MgAl_2_O_4_ catalyst, ball milled samples were calcined at 400 °C for 2 h (Figure 10). 

To synthesize the CuO/MgFe_2_O_4_ catalyst, the whole synthesis process was performed as the previous catalyst, but in the synthesis of the magnetic spinel support MgFe_2_O_4_, the MgO, and α-Fe_2_O_3_ were mixed by high speed ball milling. For other parameters such as stoichiometric ratio, calcination time and temperature, the ball milling speed and time were quite similar to the synthesis process of CuO/MgAl_2_O_4_ catalyst.

### 4.3. Characterization Techniques of the Nanocatalysts

Analysis by X-Ray Diffraction (XRD), Field Emission Scanning Electron Microscopy (FESEM), Transmission Electron Microscopy (TEM), Energy-dispersive X-ray spectroscopy (EDS) Dot mapping, X-ray Photoelectron Spectroscopy (XPS), Brunauer-Emmett-Teller (BET)/Barrett-Joyner-Halenda (BJH) and Magnetic Susceptibility techniques were used to evaluate the characteristics of the synthesized nanocatalysts. Catalyst acidity was determined by the titration method of pyridine and dimethyl pyridine through a gas chromatograph with flame ionization detector (FID) and a packed column Chromosorb AW-MCS 80/100 of 0.5 m. The surface morphology and particle size of the synthesized catalysts were analyzed by using TEM (Transmission Electron Microscopy, JEM-1400 (JEOL, Peabody, MA, USA) analyser) and SEM (Scanning Electron Microscopy, JSM-7800F Prime (JEOL, Peabody, MA, USA) analyzer). Considering the importance of the element composition on the catalyst surface, an XPS analyzer Ultra High Vacuum (UHV) multipurpose surface analysis (Specst model, Berlin, Germany) operating at pressures <10^−10^ mbar using a conventional X-ray source—XR spectra were recorded on a Bruker D8 X ray diffractometer (10–80° 2ϴ range) in the Bragg-Brentano geometry and in reflection mode, using a Cu X-ray tube, a rotating platform, a monochromatic primary beam, and a high sensitivity detector—and an EDX analyzer X-Max^N^ (OXFORD Instruments, Abingdon, UK) were applied to obtain the surface elements composition and the material types. The specific surface area and pore diameter of the catalysts were measured by using the BET-BJH method with ASAP 2000 (Micromeritics Instrument, Norcross, GA, USA) device. A MS2 A Magnetic Susceptibility Meter (Bartington Instruments, Witney, Oxon, UK) device was used to determine the magnetic power of the MgFe_2_O_4_ spinel.

### 4.4. Experimental Set-Up for the Catalytic Performance Test

Synthesized catalysts were used in the vanillin production reaction from vanillin alcohol and isoeugenol, to gain an appropriate assessment of their catalytic activity. 

The reaction was carried out in a carousel with a Pyrex tube. For the oxidation reaction of isoeugenol, 8 mL acetonitrile, 1.2 mL H_2_O_2_ (hydrogen peroxide) solution (20 mmol H_2_O_2_), and 0.8 mL isoeugenol (5 mmol) were poured into a Pyrex tube with 0.1 g of catalyst. Products were analyzed at different time intervals by a Gas chromatograph (7890A model, Agilent Technologies, Santa Clara, CA, USA) fitted with a capillary column Petrocol 100 m × 0.25 mm and 0.5 μm and a flame ionization detector (FID). The results were finally confirmed by GC-MS.

Also, for the vanillin alcohol oxidation reaction, 8 mL of acetonitrile was poured into a carousel tube and the temperature was increased to 40 °C to provide suitable conditions for complete dissolution of vanillyl alcohol. Then 0.77 g (5 mmol) of vanillyl alcohol was added to the solvent and after complete dissolution of vanillyl alcohol in acetonitrile, 1.2 mL of hydrogen peroxide with 0.1 g catalyst were added to the reaction mixture. The carousel was adjusted to 90 °C with a magnetic stirrer speed of 800 rpm. To evaluate the progress of the reactions, the reaction mixture was sampled at different times. Sampling was done by a syringe with a filter. To study the reusability of the samples, catalysts were separated after reaction by a paper filter and washed by acetonitrile. Then, they were oven dried at 110 °C for 24 h and re-used in the vanillin production reaction. To obtain conversion and selectivity of the reaction, products were analyzed by the same gas chromatograph and also, results were confirmed by GC-MS.

## Figures and Tables

**Table 1:** Structural properties of CuO/MgAl_2_O_4_ and CuO/MgFe_2_O_4_ nanocatalysts.

Spinel	Surface Area (m^2^/g)	Pore Volume (cm^3^/g)	Mean Pore Size (nm)	Crystallite Size (nm)
CuO/MgAl_2_O_4_	20	0.09	18.2	25.9 ^a^
CuO/MgFe_2_O_4_	<5	0.03	51.4	78.0 ^b^

^a^ Crystallite size estimated by Scherrer’s equation at 2θ = 36.8°. ^b^ Crystallite size estimated by Scherrer’s equation at 2θ = 35.6°.

**Table 2:** Acidity and magnetic properties of CuO/MgAl_2_O_4_ and CuO/MgFe_2_O_4_ nanocatalysts.

Nanocatalyst	Magnetic Susceptibility (10^−6^ m^3^/Kg)	Total Acidity (µmol PY/g)	Brönsted Acidity (µmol DMPY/g) ^1^	Lewis Acidity (µmol PY/g) ^2^
**CuO/MgAl_2_O_4_**	-	69	43	26
**CuO/MgFe_2_O_4_**	416	38	12	26

^1^ DMPY: 2,6-dimethylpyridine; ^2^ PY: Pyridine.

**Table 3:** Catalytic activity of CuO/MgAl_2_O_4_ and CuO/MgFe_2_O_4_ catalysts in the oxidation of isoeugenol to vanillin.

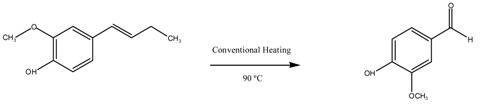					
Entry	Catalyst	Time (h)	Conversion (%mol)	Selectivity	
				Vanillin	Others
1	Blank	2	18	7	93
2	CuO/MgAl_2_O_4_	2	67	74	26
3	1 Reuse CuO/MgAl_2_O_4_	2	70	76	24
4	2 Reuse CuO/MgAl_2_O_4_	2	63	64	36
5	3 Reuse CuO/MgAl_2_O_4_	2	40	42	58
6	4 Reuse CuO/MgAl_2_O_4_	2	29	28	72
7	Blank	5	20	11	89
8	CuO/MgFe_2_O_4_	5	53	46	54
9	1 Reuse CuO/MgFe_2_O_4_	5	50	45	55
10	2 Reuse CuO/MgFe_2_O_4_	5	29	23	77
11	3 Reuse CuO/MgFe_2_O_4_	5	17	9	91
12	4 Reuse CuO/MgFe_2_O_4_	5	17	8	92
Reaction Conditions: 8 mL acetonitrile, 5 mmol isoeugenol, 20 mmol H_2_O_2_, 100 mg catalyst, 90 °C.					

**Table 4:** Catalytic activity of CuO/MgAl_2_O_4_ and CuO/MgFe_2_O_4_ catalysts in the oxidation of vanillin alcohol to vanillin.

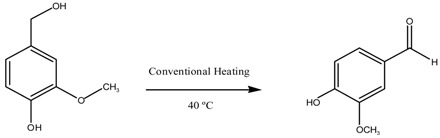					
Entry	Catalyst	Time (h)	Conversion (%mol)	Selectivity	
				Vanillin	Others
1	Blank	8	21	0	100
2	CuO/MgAl_2_O_4_	8	81	100	-
3	1 Reuse CuO/MgAl_2_O_4_	8	84	100	-
4	2 Reuse CuO/MgAl_2_O_4_	8	79	100	-
5	3 Reuse CuO/MgAl_2_O_4_	8	56	100	-
6	4 Reuse CuO/MgAl_2_O_4_	8	29	100	-
7	Blank	8	21	0	100
8	CuO/MgFe_2_O_4_	8	64	100	-
9	1 Reuse CuO/MgFe_2_O_4_	8	65	100	-
10	2 Reuse CuO/MgFe_2_O_4_	8	42	100	-
11	3 Reuse CuO/MgFe_2_O_4_	8	26	100	-
12	4 Reuse CuO/MgFe_2_O_4_	8	25	100	-
Reaction Conditions: 8 mL acetonitrile, 5 mmol vanillin alcohol, 20 mmol H_2_O_2_, 100 mg catalyst, 40 °C					

## References

[1] Shimoni E., Ravid U., Shoham Y. (2000). Isolation of a Bacillus sp. capable of transforming isoeugenol to vanillin. J. Biotechnol..

[2] Wu W., Yang L., Zhao F., Zeng B. (2017). A vanillin electrochemical sensor based on molecularly imprinted poly(1-vinyl-3-octylimidazole hexafluoride phosphorus)−multi-walled carbon nanotubes@polydopamine–carboxyl single-walled carbon nanotubes composite. Sens. Actuators B Chem..

[3] Mota M.I.F., Rodrigues Pinto P.C., Loureiro J.M., Rodrigues A.E. (2016). Recovery of Vanillin and Syringaldehyde from Lignin Oxidation: A Review of Separation and Purification Processes. Sep. Purif. Rev..

[4] Augugliaro V., Camera-Roda G., Loddo V., Palmisano G., Palmisano L., Parrino F., Puma M.A. (2012). Synthesis of vanillin in water by TiO2 photocatalysis. Appl. Catal. B Environ..

[5] Ramachandra Rao S., Ravishankar G.A. (2000). Vanilla flavour: Production by conventional and biotechnological routes. J. Sci. Food Agric..

[6] Walton N.J., Mayer M.J., Narbad A. (2003). Vanillin. Phytochemistry.

[7] Gusevskaya E.V., Menini L., Parreira L.A., Mesquita R.A., Kozlov Y.N., Shul’pin G.B. (2012). Oxidation of isoeugenol to vanillin by the “H2O2–vanadate–pyrazine-2-carboxylic acid” reagent. J. Mol. Catal. A Chem..

[8] Hua D., Ma C., Song L., Lin S., Zhang Z., Deng Z., Xu P. (2007). Enhanced vanillin production from ferulic acid using adsorbent resin. Appl. Microbiol. Biotechnol..

[9] Oddou J., Stentelaire C., Lesage-Meessen L., Asther M., Colonna Ceccaldi B. (1999). Improvement of ferulic acid bioconversion into vanillin by use of high-density cultures of Pycnoporus cinnabarinus. Appl. Microbiol. Biotechnol..

[10] Priefert H., Rabenhorst J., Steinbüchel A. (2001). Biotechnological production of vanillin. Appl. Microbiol. Biotechnol..

[11] Shiba T., Xiao L., Miyakoshi T., Chen C.L. (2000). Oxidation of isoeugenol and coniferyl alcohol catalyzed by laccases isolated from Rhus vernicifera Stokes and Pycnoporus coccineus. J. Mol. Catal. B Enzym..

[12] Ma X.-K., Daugulis A.J. (2014). Effect of bioconversion conditions on vanillin production by *Amycolatopsis* sp. ATCC 39116 through an analysis of competing by-product formation. Bioprocess Biosyst. Eng..

[13] Zhao L.-Q., Sun Z.-H., Zheng P., He J.-Y. (2006). Biotransformation of isoeugenol to vanillin by Bacillus fusiformis CGMCC1347 with the addition of resin HD-8. Process Biochem..

[14] Hua D., Ma C., Lin S., Song L., Deng Z., Maomy Z., Zhang Z., Yu B., Xu P. (2007). Biotransformation of isoeugenol to vanillin by a newly isolated Bacillus pumilus strain: Identification of major metabolites. J. Biotechnol..

[15] Dignum M.J.W., Kerler J., Verpoorte R. (2001). Vanilla production: Technological, chemical, and biosynthetic aspects. Food Rev. Int..

[16] Rodrigues Pinto P.C., Borges da Silva E.A., Rodrigues A.E., Baskar C., Baskar S., Dhillon R.S. (2012). Lignin as Source of Fine Chemicals: Vanillin and Syringaldehyde. Biomass Conversion: The Interface of Biotechnology, Chemistry and Materials Science.

[17] Ramana S., Rao B.G., Venkataswamy P., Rangaswamy A., Reddy B.M. (2016). Nanostructured Mn-doped ceria solid solutions for efficient oxidation of vanillyl alcohol. J. Mol. Catal. A Chem..

[18] Behling R., Chatel G., Valange S. (2017). Sonochemical oxidation of vanillyl alcohol to vanillin in the presence of a cobalt oxide catalyst under mild conditions. Ultrason. Sonochem..

[19] Saha S., Hamid S.B.A., Ali T.H. (2017). Catalytic evaluation on liquid phase oxidation of vanillyl alcohol using air and H2O2 over mesoporous Cu-Ti composite oxide. Appl. Surf. Sci..

[20] Wang C., Wen C., Lauterbach J., Sasmaz E. (2017). Superior oxygen transfer ability of Pd/MnOx-CeO_2_ for enhanced low temperature CO oxidation activity. Appl. Catal. B Environ..

[21] Xie S., Liu Y., Deng J., Zhao X., Yang J., Zhang K., Han Z., Dai H. (2016). Three-dimensionally ordered macroporous CeO_2_-supported Pd@Co nanoparticles: Highly active catalysts for methane oxidation. J. Catal..

[22] Jin M., Kim J.W., Kim J.M., Jurng J., Bae G.-N., Jeon J.-K., Park Y.-K. (2011). Effect of calcination temperature on the oxidation of benzene with ozone at low temperature over mesoporous α-Mn_2_O_3_. Powder Technol..

[23] Zhang X., Li H., Hou F., Yang Y., Dong H., Liu N., Wang Y., Cui L. (2017). Synthesis of highly efficient Mn_2_O_3_ catalysts for CO oxidation derived from Mn-MIL-100. Appl. Surf. Sci..

[24] Xu J., Deng Y.-Q., Luo Y., Mao W., Yang X.-J., Han Y.-F. (2013). Operando Raman spectroscopy and kinetic study of low-temperature CO oxidation on an α-Mn_2_O_3_ nanocatalyst. J. Catal..

[25] González-Cobos J., Horwat D., Ghanbaja J., Valverde J.L., de Lucas-Consuegra A. (2014). Electrochemical activation of Au nanoparticles for the selective partial oxidation of methanol. J. Catal..

[26] Saqlain M.A., Hussain A., Siddiq M., Leitão A.A. (2016). A DFT+U study of the Mars Van Krevelen mechanism of CO oxidation on Au/TiO_2_ catalysts. Appl. Catal. A Gen..

[27] Jiang W., Pang Y., Gu L., Yao Y., Su Q., Ji W., Au C.-T. (2017). Structurally defined SnO_2_ substrates, nanostructured Au/SnO_2_ interfaces, and their distinctive behavior in benzene and methanol oxidation. J. Catal..

[28] Deng Q.-F., Ren T.-Z., Agula B., Liu Y., Yuan Z.-Y. (2014). Mesoporous Ce_*x*_Zr_1−*x*_O_2_ solid solutions supported CuO nanocatalysts for toluene total oxidation. J. Ind. Eng. Chem..

[29] Menon U., Galvita V.V., Constales D., Alexopoulos K., Yablonsky G., Marin G.B. (2015). Microkinetics for toluene total oxidation over CuO–CeO_2_/Al_2_O_3_. Catal. Today.

[30] Ma Y., Li H., Wang R., Wang H., Lv W., Ji S. (2015). Ultrathin willow-like CuO nanoflakes as an efficient catalyst for electro-oxidation of hydrazine. J. Power Sources.

[31] Sun S., Mao D., Yu J. (2015). Enhanced CO oxidation activity of CuO/CeO_2_ catalyst prepared by surfactant-assisted impregnation method. J. Rare Earths.

[32] Rahmani Vahid B., Haghighi M. (2017). Biodiesel production from sunflower oil over MgO/MgAl_2_O_4_ nanocatalyst: Effect of fuel type on catalyst nanostructure and performance. Energy Convers. Manag..

[33] Dehghani F., Hashemian S., Shibani A. (2017). Effect of calcination temperature for capability of MFe_2_O_4_ (M = Co, Ni and Zn) ferrite spinel for adsorption of bromophenol red. J. Ind. Eng. Chem..

[34] Bhaduri S., Bhaduri S.B. (2002). Microstructural and mechanical properties of nanocrystalline spinel and related composites. Ceram. Int..

[35] Ganesh I., Bhattacharjee S., Saha B.P., Johnson R., Rajeshwari K., Sengupta R., Ramana Rao M.V., Mahajan Y.R. (2002). An efficient MgAl_2_O_4_ spinel additive for improved slag erosion and penetration resistance of high-Al_2_O_3_ and MgO–C refractories. Ceram. Int..

[36] Chaudhary V., Zhong Y., Parmar H., Sharma V., Tan X., Ramanujan R.V. (2018). Mechanochemical Synthesis of Iron and Cobalt Magnetic Metal Nanoparticles and Iron/Calcium Oxide and Cobalt/Calcium Oxide Nanocomposites. ChemistryOpen.

[37] Bergmann I., Šepelák V., Becker K.D. (2006). Preparation of nanoscale MgFe_2_O_4_ via non-conventional mechanochemical route. Solid State Ion..

[38] Abdi M.S., Ebadzadeh T., Ghaffari A., Feli M. (2015). Synthesis of nano-sized spinel (MgAl_2_O_4_) from short mechanochemically activated chloride precursors and its sintering behavior. Adv. Powder Technol..

[39] Scherrer P. (1918). Bestimmung der Grösse und der inneren Struktur von Kolloidteilchen mittels Röntgenstrahlen. Nachr. Ges. Wiss. Zu Gott..

[40] Rahmani Vahid B., Haghighi M., Alaei S., Toghiani J. (2017). Reusability enhancement of combustion synthesized MgO/MgAl_2_O_4_ nanocatalyst in biodiesel production by glow discharge plasma treatment. Energy Convers. Manag..

[41] Charghand M., Haghighi M., Saedy S., Aghamohammadi S. (2014). Efficient hydrothermal synthesis of nanostructured SAPO-34 using ultrasound energy: Physicochemical characterization and catalytic performance toward methanol conversion to light olefins. Adv. Powder Technol..

[42] Zhang C., Li S., Wu G., Gong J. (2014). Synthesis of stable Ni-CeO_2_ catalysts via ball-milling for ethanol steam reforming. Catal. Today.

[43] Li J., Li F., Hu K. (2004). Preparation of Ni/Al_2_O_3_ nanocomposite powder by high-energy ball milling and subsequent heat treatment. J. Mater. Process. Technol..

[44] Gao J., Yang S., Li N., Meng L., Wang F., He H., Sun C. (2016). Rapid degradation of azo dye Direct Black BN by magnetic MgFe_2_O_4_-SiC under microwave radiation. Appl. Surf. Sci..

[45] Yin Y., Liu W., Huo N., Yang S. (2017). High rate capability and long cycle stability of Fe2O3/MgFe_2_O_4_ anode material synthesized by gel-cast processing. Chem. Eng. J..

[46] Shen Y., Wu Y., Li X., Zhao Q., Hou Y. (2013). One-pot synthesis of MgFe_2_O_4_ nanospheres by solvothermal method. Mater. Lett..

[47] Tabaza W.A.I., Swart H.C., Kroon R.E. (2014). Luminescence of Ce doped MgAl_2_O_4_ prepared by the combustion method. Phys. B.

[48] Strohmeier B.R. (1994). Magnesium Aluminate (MgAl_2_O_4_) by XPS. Surf. Sci. Spectra.

[49] Tian N., Zhou Z., Tian X., Yang C., Li Y. (2017). Superior capability of MgAl_2_O_4_ for selenite removal from contaminated groundwater during its reconstruction of layered double hydroxides. Sep. Purif. Technol..

[50] Fu P., Xu Y., Shi H., Zhang B., Ruan X., Lu W. (2014). The effect of annealing process on the optical and microwave dielectric properties of transparent MgAl_2_O_4_ ceramics by spark plasma sintering. Opt. Mater..

[51] Elmhamdi A., Castañeda R., Kubacka A., Pascual L., Nahdi K., Martínez-Arias A. (2016). Characterization and catalytic properties of CuO/CeO_2_/MgAl_2_O_4_ for preferential oxidation of CO in H2-rich streams. Appl. Catal. B Environ..

[52] Fernández-García M., Gómez Rebollo E., Guerrero Ruiz A., Conesa J.C., Soria J. (1997). Influence of Ceria on the Dispersion and Reduction/Oxidation Behaviour of Alumina-Supported Copper Catalysts. J. Catal..

[53] Martínez-Arias A., Fernández-García M., Soria J., Conesa J.C. (1999). Spectroscopic Study of a Cu/CeO_2_Catalyst Subjected to Redox Treatments in Carbon Monoxide and Oxygen. J. Catal..

[54] Gamarra D., Munuera G., Hungría A.B., Fernández-García M., Conesa J.C., Midgley P.A., Wang X.Q., Hanson J.C., Rodríguez J.A., Martínez-Arias A. (2007). Structure—Activity Relationship in Nanostructured Copper—Ceria-Based Preferential CO Oxidation Catalysts. J. Phys. Chem. C.

[55] Campelo J.M., Luna D., Luque R., Marinas J.M., Romero A.A., Calvino J.J., Rodríguez-Luque M.P. (2005). Synthesis of acidic Al-MCM-48: Influence of the Si/Al ratio, degree of the surfactant hydroxyl exchange, and post-treatment in NH4F solution. J. Catal..

[56] Gracia M.J., Losada E., Luque R., Campelo J.M., Luna D., Marinas J.M., Romero A.A. (2008). Activity of Gallium and Aluminum SBA-15 materials in the Friedel–Crafts alkylation of toluene with benzyl chloride and benzyl alcohol. Appl. Catal. A Gen..

[57] Luque R., Campelo J.M., Luna D., Marinas J.M., Romero A.A. (2005). NH4F effect in post-synthesis treatment of Al-MCM-41 mesoporous materials. Microporous Mesoporous Mater..

[58] Pineda A., Balu A.M., Campelo J.M., Luque R., Romero A.A., Serrano-Ruiz J.C. (2012). High alkylation activities of ball-milled synthesized low-load supported iron oxide nanoparticles on mesoporous aluminosilicates. Catal. Today.

